# Genetic and epigenetic mechanisms in the development of arteriovenous malformations in the brain

**DOI:** 10.1186/s13148-016-0248-8

**Published:** 2016-07-22

**Authors:** Jaya Mary Thomas, Sumi Surendran, Mathew Abraham, Arumugam Rajavelu, Chandrasekharan C. Kartha

**Affiliations:** Cardiovascular Disease Biology Program, Rajiv Gandhi Centre for Biotechnology, Poojapura, Thycaud, Thiruvananthapuram, Kerala India; Tropical Disease Biology Program, Rajiv Gandhi Centre for Biotechnology, Poojapura, Thycaud, Thiruvananthapuram, Kerala India; Department of Neurosurgery, Sree Chitra Tirunal Institute for Medical Sciences & Technology, Thiruvananthapuram, Kerala India

## Abstract

Vascular malformations are developmental congenital abnormalities of the vascular system which may involve any segment of the vascular tree such as capillaries, veins, arteries, or lymphatics. Arteriovenous malformations (AVMs) are congenital vascular lesions, initially described as “erectile tumors,” characterized by atypical aggregation of dilated arteries and veins. They may occur in any part of the body, including the brain, heart, liver, and skin. Severe clinical manifestations occur only in the brain. There is absence of normal vascular structure at the subarteriolar level and dearth of capillary bed resulting in aberrant arteriovenous shunting. The causative factor and pathogenic mechanisms of AVMs are unknown. Importantly, no marker proteins have been identified for AVM. AVM is a high flow vascular malformation and is considered to develop because of variability in the hemodynamic forces of blood flow. Altered local hemodynamics in the blood vessels can affect cellular metabolism and may trigger epigenetic factors of the endothelial cell. The genes that are recognized to be associated with AVM might be modulated by various epigenetic factors. We propose that AVMs result from a series of changes in the DNA methylation and histone modifications in the genes connected to vascular development. Aberrant epigenetic modifications in the genome of endothelial cells may drive the artery or vein to an aberrant phenotype. This review focuses on the molecular pathways of arterial and venous development and discusses the role of hemodynamic forces in the development of AVM and possible link between hemodynamic forces and epigenetic mechanisms in the pathogenesis of AVM.

## Background

International Society for the Study of Vascular Anomalies (ISSVA) has categorized vascular anomalies into two primary biological categories: (1) vascular neoplasms and (2) vascular malformations [1]. Vascular neoplasms are characterized by increased endothelial cell turn over whereas vascular malformations are not [2]. Vascular malformations are structural abnormalities which affect any part of the vascular system such as artery, vein, capillary or lymphatics. They can occur in any part of the human body and can be present at any age [3]. Arteriovenous malformation (AVM) was initially described and classified as a separate pathological entity in mid-1800s by Luschka (1854) and Virchow (1863) [4, 5]. Surgical exposure of AVM was first performed by Giordano in the year 1889; later, Green and Vaugham in the year 1972 initiated the era of microsurgery treatment for AVM [6, 7]. There have been several attempts to classify vascular malformation based on the anatomic appearance, arteriovenous shunting, hemodynamics, and contrast angiographic appearance, but the complexity in disease status had made classification of vascular malformations very difficult [8–11]. Hamburg classification system categorized vascular malformations based on the predominant vascular defect whether it is arterial, venous, arteriovenous, lymphatic, or combined. This classification system is currently widely accepted by pathologists [12]. Although vascular malformations may develop in any part of the body, the cerebral vascular malformations are the most severe. They are divided into four types: arteriovenous malformations, developmental venous anomalies, cavernous malformations, and capillary telangiectasias [13]. This review focuses on the molecular mechanisms in the development of cerebral arteriovenous malformations.

## Structural anomalies in arteriovenous malformations

**Fig. 1:**
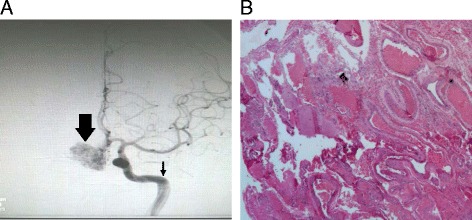
**a** Cerebral angiogram of an AVM in frontal lobe of the brain. Nidus (*thick arrow*) and feeding artery (*thin arrow*). **b** Photomicrograph of an AVM in the temporal lobe of the brain (hematoxylin-eosin ×4)

Arteriovenous malformations are high-flow vascular malformations characterized by direct contact of arteries to veins without an intervening capillary bed. AVM consists of three components: a group of anomalous blood vessels (nidus), a feeding artery, and a draining vein. Feeding artery could be from anterior (internal carotid, anterior cerebral, middle cerebral), posterior (posterior cerebral, vertebra basilar system), or extra cranial vessels, and there can be single or multiple feeding arteries. Based on the hemodynamic contribution to AVM, it can be a dominant feeder or a supplementary feeder. AVM is often characterized by a single large draining vein, though multiple draining veins can also be present. Draining vein can be either superficial or deep centered [12–14]. Anomalous blood vessels are grossly divided into three groups: type A, resembles an arterial structure with duplication of elastic lamina and reduced or scanty medial muscular layer; type B, similar to hypertrophic or degenerative veins with scarcity of internal elastic lamina or elastic fibers; and type C, consists of vessels less than 150 μm in diameter intervened between type A and type B vessels with a prominent increase in elastic fibers [15] (Fig. 1b). Lesions of AVM can be either focal or diffused. Focal lesions are highly compact with no intervening cerebral parenchyma, whereas diffused lesions have normal cerebral tissue entrapped between blood vessels [14]. Historical concept on the congenital nature of AVM is challenged by the recurrence of AVM, even after complete surgical removal [16–22].

There is no sex difference prediction for AVM incidence, and the clinical course of the disease is similar in both males and females [23]. Cerebral hemorrhage is the common presenting symptom which leads to diagnosis of AVM. Epileptic fits could also be a presenting symptom. Epileptic fits could be seen even 9 years before AVM is diagnosed [16–24]. Other presenting signs of AVM include headaches, asymptomatic bruits, migraine, psychiatric disorders, visual field defects, dizziness, balance disturbances, and migraine [25, 26]. AVMs can occur in any lobe of the brain; the majority of AVMs are supratentorial with the parietal lobe being frequently affected. The size of the AVM can vary from 2 to 6 cm. The small and deep-centered AVMs are more prone to rupture than large AVMs [24, 25]. Computed tomographic angiography, magnetic resonance angiography, and cerebral angiography are used to visualize AVM (Fig. 1a). There are several modalities available for the management of AVM. They include stereotactic radiotherapy, microsurgical resection, and endovascular embolization; operative treatment is most preferred to prevent the intermittent risk of intracranial hemorrhage and for complete eradication of the disease [27].

## Molecular basis of AVM pathogenesis

Our understanding about AVM and its nature of development in the brain are evolving rapidly. The lesions are no longer considered as static lesions. They are highly dynamic with features of rapid growth, remodeling, regression, and de novo formation even after complete surgical removal [16–22, 28]. Two basic approaches have been followed to identify the genetic basis of AVMs in humans: (i) the family linkage analysis in patients with AVM and (ii) analysis of the defective genes using post-operative specimens [14]. In most cases, it has been observed that aberrant molecular signaling in the normal vasculogenesis process leads to vascular malformations. AVM as congenital lesions is associated with hereditary hemorrhagic telangiectasia (HHT), Wyburn-Mason syndrome, Osler-Weber-Rendu disease, and Sturge-Weber syndrome [29–33]. The importance of single nucleotide polymorphisms in several genes linked to angiogenesis and inflammation and their association for AVM development have been reviewed [34]. The presence of mutations in the genes of endoglin and activin receptor-like kinase 1 predisposes to aberrant TGF-beta signaling and is associated with HHT. These mutations commonly lead to loss of function in the genes associated with TGF-beta signaling [35]. It has also been shown that genes that code for angiopoietins (ANGPT1 and ANGPT2) and their receptor TIE-2 have a crucial role in angiogenesis and vascular stability and there are reports available that in AVM, there is an imbalance of TIE-2-angiopoietin system [36].

## Development of blood vessels

Anatomists long ago have demonstrated morphological differences between arteries and veins. The primary anatomical difference is that veins have thin walls with valves whereas arteries have thick muscular walls and do not have any valves. Functionally, they differ as arteries carry oxygenated blood whereas veins carry deoxygenated blood except in pulmonary circulation where it is reversed [37]. During development, how the endothelial cells are committed to be an artery or a vein or a capillary is a subject of much debate. The morphological features of artery and vein are thought to be acquired during developmental stages on exposure to various hemodynamic forces. Recent advances in vascular and molecular biology have identified that hemodynamic forces as well as genetic factors have significant roles in determining the fate of an endothelial cell, either of an artery or a vein. Studies on vascular development using various experimental models have identified unique molecular markers expressed by arteries and veins and those that are vital to maintain their identity [38–48] (Table 1). Some studies have also focussed on microenvironment that is important in governing venous/arterial identity [49, 50]. The genetic factors which determine the arterial and venous endothelial cell fate was first understood with the discovery of Ephrin family members. From recent reports on molecular mechanisms for specification of arteriovenous identity, it is evident that EFNB2 and EPHB4 markers are the end determinants of arterial and vein specification, respectively. Significance of EFNB2/EPHB4 signaling in arteriovenous specification was first recognized by Wang HU, and their study concluded that morphological differences between arteries and veins are partly determined by genetic factors [51]. It is now known that shear stress activates EFNB2 expression in murine embryonic stem cells through vascular endothelial growth factor (VEGF)-Notch signaling [52]. Thus, it is evident that VEGF-Notch signaling pathway determines the arterial specification and is controlled by hemodynamic forces. The molecular distinction established by EFNB2/EPHB4 are not a temporary characteristics of the developing vascular system but have their impact on adult arteries and veins as well [44]. The coordinated action of Hedgehog-VEGF-Notch signaling cascade is essential to control the arterial/venous fate during development. Recent studies have revealed that VEGF acts downstream of Sonic Hedgehog and upstream of the Notch pathway and decides arterial endothelial cell fate [53]. The role of Hedgehog-VEGF-Notch signaling in controlling arterial endothelial cell fate is more evident with identification of calcitonin receptor-like receptor (CALCRL) as a novel element in Hedgehog-VEGF-Notch signaling cascade. Somitic CALCRL expression is in turn regulated by sonic hedgehog. Downregulation of CALCRL affects VEGF expression, and CALCRL morphants are characterized by the absence of expression of arterial markers like EFNB2, Delta C, and Notch5 [54]. Notch signaling pathway plays a pivotal role in differentiation of endothelial cell to artery. Downregulation of Notch leads to decreased expression of arterial specific markers EFNB2 and NOTCH3, along with constitutive increase of venous markers like FLT4 and RTK5 [55]. Simultaneous activation of Notch along with β-catenin promotes arterial endothelial cell formation from Flk1vascular progenitors and maintains the arterial endothelial cell phenotype [56]. Notch signaling pathway in association with EFNB2/EPHB4 determines the size of the blood vessel as well as proportion of arterial and venous endothelial cells [57]. The Role of Notch in arterial specification is further clarified by the discovery of gridlock signaling which acts downstream of Notch. In zebrafish, downregulation of gridlock expression transforms arterial phenotype to venous [58]. It is evident that the cumulative actions of molecular signaling and hemodynamic forces are crucial for the proper development and maintenance of arteries and veins; any dysregulation in these actions may lead to the development of AVMs.

**Table 1:** Known vascular markers specific for arteries and veins

S. no	Arterial markers	Venous markers
1	EFNB2	EPHB4
2	Delta-like 4 (DLL4)	Neuropilin 2 (NRP2)
3	Activin-receptor-like kinase 1 (ACVRL1)	COUP-TFII
4	Endothelial PAS domain protein 1 (EPAS1)	FLT4
5	HEY1	RTK5
6	HEY2	
7	Neuropilin 1 (NRP1)	

## Do hemodynamic forces modulate the epigenetic landscape in AVM?

**Fig. 2:**
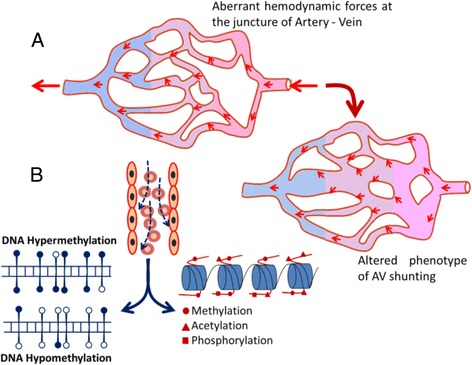
**a** Schematic representation of how aberrant hemodynamic forces cause altered arteriovenous shunting and further develops into AVM. **b** The altered blood forces may result in aberrant epigenetic landscape with possibility of hypo- or hyper-DNA methylations and alterations of various histone modifications and lead to AVM

Both genetic and epigenetic factors determine the identity of endothelial cell in an artery or a vein or a capillary. Hove et al. (2003) showed that intracardiac fluid forces regulate the various epigenetic factors during embryonic cardiogenesis. Their study established the fact that in response to flow-induced forces, cultured cardiac endothelial cells remodel their cytoskeletal structures and change their gene expression profiles [59]. Connection between hemodynamic forces and epigenetic modulators were studied in detail by Illi et al. They reported the exposure of endothelial cells to shear stress results in a series of chromatin modifications which causes changes in gene expression in these cells [60]. The causes for development of AVMs in the human brain are still elusive. It is widely speculated that altered hemodynamic forces at the junction of an artery and a vein might provoke the development of AVM and hemodynamic forces may be the critical epigenetic regulator in deciding the endothelial fate [61]. AVM is a high-flow vascular abnormality, and cerebral hemodynamic flow changes have an important role in AVM pathophysiology, which includes hemorrhage, ischemia, and seizures [62, 63]. Two important hemodynamic parameters like blood pressure and wall shearing stress induce vascular remodeling. It has been described that immense wall shearing stress and circumferential strain in AVM feeders activate endothelial changes, which results in increased expression of factors like matrix metalloprotease 9, platelet-derived growth factor, and VEGF that eventually induce vascular remodeling in humans [64–68]. Studies in activin receptor-like kinase 1 deficient mice model suggest that change in the wall shearing stress induces higher expression of VEGF, which leads to the formation of distended vessels in the mouse brain. The vascular morphological features in activin receptor-like kinase 1 deficient mice are very similar to those of AVMs in humans [69, 70]. All existing studies suggest that the abrupt changes in the hemodynamic flow in the junction of arteriovenous blood vessels might strongly influence the epigenetic mediators and eventually lead to the development of AVM (Fig. 2). It has been reported that disturbed shear pressure and altered hemodynamic forces cause endothelial dysfunction at the arterial branches and curvatures of blood vessels [71]. In addition, disrupted shear and oscillatory hemodynamic pressure activates many atherogenic genes in endothelial cells and promotes atherosclerosis [72]. Various epigenetic factors could be involved in the development of AVM due to variations in hemodynamic forces.

## DNA methylation and methyltransferases in AVM

The development of cells and their differentiation are controlled by several epigenetic regulators. DNA methylation is a key mechanism of epigenetic regulation in higher eukaryotes. The DNA methylation reaction is catalyzed by a group of enzymes called DNA methyltransferases which uses S-adenosyl-l-methionine as methyl donor [73]. In humans, there are two groups of DNA methyltransferases, DNMT3A and DNMT3B. These enzymes are de novo DNA methyltransferases and set the initial DNA methylation patterns during development. DNMT1 enzyme copies the DNA methylation pattern from parental strand to daughter strand during DNA replication [74, 75]. The methylation of CpG sites at gene promoter causes the stable silencing of gene expression, and it is essential during early embryonic development. These DNA methylation marks are important for the establishment of totipotency or pluripotency as well as for health later in life [76]. In addition, DNA methylation has a pivotal role in X-chromosome inactivation, maintaining cell pluripotency, genomic imprinting, and cellular integrity. Aberrant DNA methylation pattern is a well-characterized epigenetic hallmark in several pathologies particularly in many cancers. It is known that aberrant expression of DNMTs and aberrant DNA methylation signatures occur in various cancers, cardiac development, and vascular developmental diseases [77, 78]. The presence of hypomethylation at global level with increased transcriptional activity in endothelial cells of artery during atherogenesis process suggests that local stimuli in endothelial cells of an artery can bring changes at DNA methylation level in their genome [79]. It has been shown that the differential methylation of promoter of endothelial nitric oxide synthase (eNOS) gene contributes to vascular function, indicating DNA methylation plays a significant role in maintaining endothelial cell function [80]. It is known that the artery and vein are functionally and morphologically different; importantly, their endothelial cells have marked variation in the level of DNA methylations in their genome. Recently, Joo et al. reported the presence of differential methylation between artery and venous endothelial cell types [81]. They also observed inverse relation between gene expression and promoter methylation of a subset of genes and that such DNA methylation pattern functionally reflected in endothelial cell function [81]. The MeCP2 and ubiquitin-like PHD and RING finger domain-containing proteins are methyl-binding proteins, known to interact with the DNMTs and regulate their activity. The methyl-binding proteins bind to 5-methyl cytosine with high affinity and further modulate the local gene activity [82]. It has been shown that in vascular endothelial cells, methyl-binding proteins bind to methylated promoters of eNOS and vascular endothelial growth factor receptor 2 (VEGFR2) [83]. Ablation of methyl-binding proteins in these cells leads to activation of eNOS and VEGFR2 gene expression and stimulates proangiogenetic signal pathway. Therefore, it is obvious that DNA methylation and binding of its effector proteins to methylated DNA is essential for vascular function and their development [83]. The presence of somatic mutations in various epigenetic proteins has been observed in acute myeloid leukemia and leads to the development of aberrant epigenetic landscape [84, 85]. Thus, we speculate the presence of aberrant epigenetic landscape in AVM, particularly acquired mutations in epigenetic proteins and alterations of DNA methylations in the promoters of vascular developmental specific pathway genes (Fig. 2). A detailed study on the DNA methylome of AVM tissues would help us to delineate the aberrant vascular pathway genes associated with AVMs.

## Chromatin modifiers in AVMs

The N-terminus of histone tails in eukaryotes is subjected to many post-translational modifications. Among these, lysine acetylation and lysine methylation are the major modifications which regulate chromatin structure and local gene activity [86]. Epigenetic modifications in the histone tails of endothelial cells in various physiological conditions are the central theme of this review. Therefore, we have focused only acetylation and methylation of histone tails in endothelial cells. Endothelial cells are component of blood vessel, which are exposed to shear stress during aberrant blood flow condition. Shear stress a component of hemodynamic forces induces modifications at the core histones H3 and H4, particularly acetylation and phosphorylation at H3 tails, and this is a pre-requisite for shear stress-dependent expression of genes in endothelial cells [87]. Hemodynamic force-induced histone acetylation, particularly changes of its enzyme histone acetyl transferases (HATs) and histone deacetyl transferases (HDACs), has been studied in detail in the recent years [88]. It has been shown that class I HDAC1/2/3 molecules modulate the endothelial cell proliferation in response to oscillatory blood flow [89]. Disturbed blood flow changes the post-translational modifications of chromatin, which causes the changes in chromatin structure [89]. Many other studies have revealed that shear stress triggers the HDAC7 function during angiogenic process, and it is essential during differentiation of vascular endothelial cells and regenerative process [87]. There are reports that global inhibition of class I and II HDACs impairs angiogenesis process and that downregulation of HDAC7 expression impairs the development of the normal vasculature [90]. Using small interfering RNA (siRNA) approach, Mottet et al. showed that HDAC7 is crucial for the development of blood vessels. They observed that silencing of HDAC7 in endothelial cells altered the cell morphology, migration, and the capacity of cells to form capillary tube-like structures [90]. Although many studies have explored the role of histone acetylation in vascular development, there are no reports regarding histone methylation. A detailed enquiry is ***therefore warranted*** for analysis of methylation at H3 tail at K4, K9, K36, and H4 tail K20 positions in endothelial cells under various hemodynamics conditions. Methylation at these positions largely influences the gene activity, and we hypothesize that hemodynamic blood flow could affect these methylation marks and alter the epigenetic landscape and chromatin structure in developing blood vessels. These mechanisms could be involved in the pathogenesis of AVM (Fig. 2). The role of any other epigenetic modifications and enzymes in the regulation of early and late phases of vascular development is currently unknown, and further studies are required to understand such epigenetic mechanisms in AVM development.

## Conclusions

Arteriovenous malformations are congenital developmental disorders seen at many sites in the body. Pathogenesis of AVM is still largely unknown, and no specific markers have been identified for AVM. HHT patients have symptoms similar to those in patients with AVM. In HHT patients, polymorphism in TGF-beta co-receptors endoglin and activin receptor-like kinase 1 plays critical roles in the development of the HHT lesions [29–32]. The possibility of combinatorial changes at genetic and epigenetic levels in vascular development may lead to the abnormal phenotype of AVM. It is known that extensive tissue remodeling occurs in AVM lesions and this may arise from the changes in the epigenetic landscape of genes essential for vascular development. All previous studies suggest that development of AVMs requires stimuli to endothelial cells in blood vessels, which could be either at the transcriptional level or metabolite level in the cytoplasm of endothelial cells. Altered transcription could be due to changes at epigenetic makeup or at the gene level. Changes in the metabolites in the endothelial cells may also trigger AVM development. A detailed study of the epigenetic signals of genes associated with vascular development pathways could throw light on the genetic and epigenetic mechanisms in the development of AVMs.
