# Correction to: Genetic and epigenetic mechanisms in the development of arteriovenous malformations in the brain

**DOI:** 10.1186/s13148-018-0560-6

**Published:** 2018-10-17

**Authors:** Jaya Mary Thomas, Sumi Surendran, Mathew Abraham, Arumugam Rajavelu, Chandrasekharan C. Kartha

**Affiliations:** 1 Cardiovascular Disease Biology Program, Rajiv Gandhi Centre for Biotechnology, Poojapura, Thycaud, Thiruvananthapuram, Kerala India; 2 Tropical Disease Biology Program, Rajiv Gandhi Centre for Biotechnology, Poojapura, Thycaud, Thiruvananthapuram, Kerala India; 3 Department of Neurosurgery, Sree Chitra Tirunal Institute for Medical Sciences & Technology, Thiruvananthapuram, Kerala India; 4 Manipal Academy of Higher Education (MAHE), Manipal, Karnataka India

## Correction

Upon publication of the original article [1] the authors noticed that the affiliation Manipal Academy of Higher Education (MAHE), Manipal, Karnataka, India was missing. The addition of this affiliation has been formally noted in this correction article.
